# Clinical Outcomes of Treatment-Naive Transformed vs. De Novo Diffuse Large B-Cell Lymphoma: A Propensity Score-Matched Analysis of 1735 Cases

**DOI:** 10.3390/cancers18101641

**Published:** 2026-05-19

**Authors:** Jin Chai, Wenhui Zhang, Yue Wang, Jie Chen, Yuqin Song, Hui Yu

**Affiliations:** Key Laboratory of Carcinogenesis and Translational Research (Ministry of Education), Department of Lymphoma, Peking University Cancer Hospital & Institute, Beijing 100142, China; 2411110734@bjmu.edu.cn (J.C.); 2311210662@bjmu.edu.cn (W.Z.); 2511110735@bjmu.edu.cn (Y.W.); 2511110736@bjmu.edu.cn (J.C.)

**Keywords:** diffuse large B-cell lymphoma, treatment-naive transformation, propensity score matching, prognostic stratification

## Abstract

**Background**: The prognostic significance of histological transformation (HT) in treatment-naive diffuse large B-cell lymphoma (DLBCL) remains controversial. This study aimed to evaluate the clinical outcomes and failure patterns of treatment-naive transformed DLBCL (trDLBCL) compared with de novo DLBCL using a large-scale cohort. **Methods**: We retrospectively analyzed 1735 consecutively enrolled treatment-naive DLBCL patients (118 trDLBCL and 1617 de novo). Propensity score matching (PSM) was performed to balance baseline characteristics. Survival outcomes were assessed using Kaplan–Meier and Cox proportional hazards models. Subgroups were defined by pathology (t-FL vs. t-MZL) and pattern: concurrent (synchronous indolent lymphoma and DLBCL components at diagnosis) vs. pure transformation (DLBCL occurring as the sole histology in patients with a prior history of untreated indolent lymphoma). **Results**: In the overall cohort, trDLBCL was associated with significantly inferior progression-free survival (PFS) compared with de novo disease and remained an independent adverse prognostic factor in multivariable analysis (HR 1.754, *p* < 0.001). These findings were confirmed in a 1:1 propensity score-matched cohort (108 pairs), where trDLBCL continued to show worse PFS (*p* < 0.01), while overall survival (OS) was comparable (*p* = 0.99). Within trDLBCL patients, the underlying indolent subtype (t-FL vs. t-MZL) did not significantly affect survival (PFS *p* = 0.17, OS *p* = 0.35), whereas “pure transformation” was associated with markedly inferior PFS (*p* = 0.005) and OS (HR 2.56, *p* = 0.02) compared with concurrent transformation. Failure pattern analysis revealed a higher risk of early progression in trDLBCL (POD24: 30.56% vs. 18.52%; OR 1.94, 95% CI: 1.05–3.56), whereas central nervous system (CNS) involvement was low and comparable between groups (2.78% vs. 0.93%, *p* = 0.62). **Conclusions**: Treatment-naive trDLBCL is associated with inferior PFS driven by early progression, whereas OS is comparable due to effective salvage therapies. Pure transformation appeared to define a higher-risk subgroup with inferior disease control, supporting the need for future prospective studies to evaluate risk-adapted frontline, consolidation, or maintenance strategies.

## Simple Summary

Diffuse large B-cell lymphoma (DLBCL) can arise as a new diagnosis (de novo) or through the transformation of a pre-existing indolent lymphoma (trDLBCL). Whether trDLBCL carries a worse prognosis in treatment-naive patients remains a subject of debate. In this large-scale study of 1735 patients, we used propensity score matching to compare the clinical outcomes of these two groups. Our results show that while patients with trDLBCL have a higher risk of early disease progression (POD24) and significantly shorter progression-free survival (PFS), their overall survival (OS) is comparable to that of those with de novo DLBCL, likely due to effective subsequent salvage therapies. Importantly, we identified that “pure transformation” (patients without a concurrent indolent component at diagnosis) represents an ultra-high-risk subgroup with markedly worse outcomes. These findings emphasize that trDLBCL is a distinct clinical entity requiring more intensive or risk-adapted frontline treatment strategies to prevent early relapse and overcome intrinsic therapy resistance.

## 1. Introduction

Diffuse large B-cell lymphoma (DLBCL) is the most common type of adult non-Hodgkin lymphoma [1,2,3]. While the majority of cases are de novo, a substantial subset arises from histological transformation (HT) of pre-existing indolent lymphomas [4,5,6]. In the pre-rituximab era, transformed DLBCL (trDLBCL) was associated with a poor prognosis [7,8]. Even in the immunochemotherapy era, large-scale analyses based on the SEER database indicate that survival outcomes for trDLBCL remain generally inferior to those of de novo DLBCL [9,10].

However, most prior studies have focused on “secondary transformation” occurring after multiple lines of therapy, where prognosis may be confounded by multidrug resistance due to prior chemotherapy exposure [5,10,11,12,13]. In the treatment-naïve setting, trDLBCL may be further categorized according to transformation pattern. Concurrent transformation refers to the synchronous presence of indolent lymphoma and DLBCL components at diagnosis, whereas pure transformation refers to DLBCL occurring as the sole histology in patients with a prior history of untreated indolent lymphoma [12,14,15]. Studies by Ghesquières (2006) and Magnano (2017) suggest that patients with treatment-naive transformation have better outcomes compared to those with secondary transformation, likely due to the absence of drug selection pressure on tumor cells [14,15].

The comparative prognosis between treatment-naive trDLBCL and de novo DLBCL remains uncertain [5,11,14,16]. Although several studies have reported comparable overall survival (OS), these findings may obscure clinically relevant differences in disease kinetics, particularly with respect to early disease control [11,15]. Whether a transformation background confers an increased risk of early progression—such as progression of disease within 24 months (POD24)—rather than affecting long-term survival remains unclear [5,11,15,17]. In addition, data regarding distinct patterns of treatment failure, including central nervous system (CNS) involvement, are limited and largely derived from heterogeneous cohorts without adequate adjustment for baseline imbalances [10,11,14,18,19].

To address these gaps, we conducted a large single-center study of over 1700 patients with newly diagnosed DLBCL, focusing specifically on treatment-naive transformation. By applying propensity score matching (PSM), we constructed a well-balanced cohort of treatment-naive trDLBCL and de novo DLBCL patients. We aimed to systematically evaluate the impact of transformation background on progression-free survival (PFS) and OS, with particular emphasis on POD24 and patterns of treatment failure. In addition, we explored the prognostic heterogeneity within transformed disease, including differences between transformation subtypes and patterns (e.g., pure vs. concurrent transformation), to refine risk stratification in this population.

## 2. Patients and Methods

### 2.1. Patients and Study Design

We retrospectively screened consecutive patients newly diagnosed with DLBCL at Peking University Cancer Hospital between 2012 and 2022. After applying predefined exclusion criteria, including primary central nervous system lymphoma, primary mediastinal large B-cell lymphoma, and insufficient clinical or follow-up information, 1735 eligible patients were included in the final analysis. No additional case selection was performed beyond these predefined eligibility criteria, and 118 cases of treatment-naïve trDLBCL and 1617 cases of de novo DLBCL were identified.

Treatment-naïve trDLBCL was defined in accordance with the World Health Organization (WHO) Classification of Haematolymphoid Tumours, 5th edition, section “Transformations of indolent B-cell lymphomas” (pp. 455–460) and diagnosed as DLBCL arising from a pre-existing indolent non-Hodgkin lymphoma (iNHL) without prior systemic anti-lymphoma therapy [20]. Based on transformation patterns, trDLBCL was further classified as: (1) concurrent transformation, defined by the simultaneous presence of iNHL and DLBCL components at diagnosis. This encompassed both composite cases (two components within the same biopsy specimen) and discordant cases (e.g., DLBCL in a nodal biopsy with a concurrent low-grade component identified in other sites); and (2) pure transformation, defined as DLBCL occurring as the sole histology in patients with a prior history of iNHL without prior treatment.

All diagnoses were established by experienced hematopathologists based on comprehensive clinicopathologic evaluation, including morphology, immunophenotype, clinical features, disease distribution, and available molecular or cytogenetic results. Molecular evidence of clonal relatedness was applied when available, but was not uniformly mandatory for routine diagnosis. Cases suspicious for unrelated synchronous lymphomas were excluded from the concurrent trDLBCL group.

For trDLBCL patients, the baseline for survival analyses was defined as the time of histological or clinical confirmation of transformation. Patients with missing key variables were excluded. The study was conducted in accordance with the Declaration of Helsinki and approved by the institutional ethics committee.

### 2.2. Data Collection and Endpoints

Baseline covariates collected at diagnosis included sex, age (>60 years), Ann Arbor stage, presence of B symptoms, number of extranodal sites (>1), bulky disease (≥7 cm), ECOG performance status (>1), lactate dehydrogenase (LDH) levels, β2-microglobulin levels, and cell-of-origin (COO) classification using the Hans algorithm. Follow-up was censored on 12 December 2025.

### 2.3. Statistical Analysis

Baseline characteristics were compared using the Chi-square test or Fisher’s exact test, as appropriate. Survival curves were estimated using the Kaplan–Meier method and compared with the log-rank test. Multivariable Cox proportional hazards models were used to calculate hazard ratios (HRs) and 95% confidence intervals (CIs). PSM was performed using a logistic regression model with 1:1 nearest-neighbor matching (caliper = 0.2) to minimize selection bias. A standardized mean difference (SMD) < 0.1 was considered indicative of adequate balance between groups. The risk of POD24 was evaluated using odds ratios (ORs). Statistical analyses were conducted using SPSS 26.0, R 4.2.0, and GraphPad Prism 10.1. A two-sided *p* value < 0.05 was considered statistically significant.

## 3. Results

### 3.1. Baseline Clinical Characteristics

A total of 1735 newly diagnosed DLBCL patients were included in this study, comprising 118 cases (6.8%) of trDLBCL and 1617 cases (93.2%) of de novo DLBCL. The median age of the entire cohort was 57.6 years, with 56.7% being male. No significant differences were observed between the two groups in terms of sex (*p* = 0.13), age (*p* = 0.13), or COO subtype distribution (germinal center B-cell-like/GCB subtype: 43.2% vs. 33.0%, *p* = 0.07). Although the proportion of patients with Ann Arbor stage III–IV disease was higher in the trDLBCL group (61.0% vs. 53.2%, *p* = 0.10), the incidence of bulky disease (≥7 cm) was significantly lower compared to the de novo DLBCL group (13.6% vs. 22.2%, *p* = 0.03) (Table 1).

Among trDLBCL patients, 56 (47.5%) had transformed marginal zone lymphoma (t-MZL), 55 (46.6%) had transformed follicular lymphoma (t-FL), and 7 (5.9%) had other subtypes. Regarding transformation patterns, 56 patients (47.5%) had “pure transformation” and 62 patients (52.5%) had “concurrent transformation” (Table 1).

### 3.2. HT Is an Independent Risk Factor for PFS

**Figure 1:**
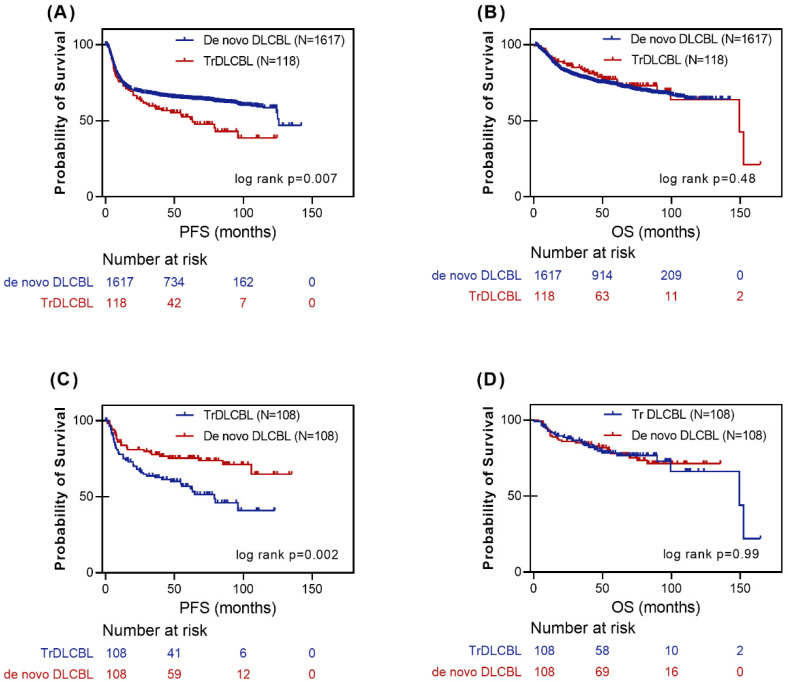
Survival Outcomes of TrDLBCL versus De Novo DLBCL. (**A**) PFS in the entire cohort. TrDLBCL patients had a significantly inferior PFS (log-rank *p* = 0.007) compared to de novo DLBCL patients. (**B**) OS in the entire cohort. There was no statistically significant difference in OS between the two groups (log-rank *p* = 0.48). (**C**) PFS in the PSM cohort. After accounting for baseline clinical differences with 1:1 matching, TrDLBCL remained associated with significantly inferior PFS (log-rank *p* = 0.002). (**D**) OS in the PSM cohort. In the balanced matched cohort, OS remained comparable between TrDLBCL and de novo DLBCL (log-rank *p* = 0.99).

Given these baseline differences, we next evaluated whether HT independently affects survival outcomes. The median follow-up for the cohort was 56.5 months. Kaplan–Meier survival analysis demonstrated that trDLBCL patients had significantly inferior PFS compared with de novo DLBCL (median PFS: 63.0 months vs. 125.6 months, log-rank *p* = 0.008). In contrast, OS did not differ significantly (median OS: 149.3 months vs. NR, log-rank *p* = 0.48) (Figure 1). In multivariable Cox regression, trDLBCL was independently associated with worse PFS (HR 1.75, 95% CI: 1.30–2.37, *p* < 0.001) (Table 2).

To account for baseline imbalances, a 1:1 nearest-neighbor PSM method was performed, yielding 108 matched pairs. Covariates were well balanced (SMD < 0.1). In the matched cohort, trDLBCL remained associated with inferior PFS (log-rank *p* < 0.01; stratified Cox HR = 2.00, *p* = 0.003), whereas OS remained comparable (*p* = 0.99) (Figure 1). These results indicate that the PFS disadvantage is attributable to transformation history rather than baseline clinical differences.

### 3.3. Impact of Transformation Patterns on Survival Outcomes

Having established that PFS remained inferior in trDLBCL after matching, we further explored whether underlying indolent subtypes or transformation patterns contributed to prognostic heterogeneity. Within trDLBCL patients, the indolent subtype (t-FL vs. t-MZL) did not significantly influence PFS (*p* = 0.17) or OS (*p* = 0.63), suggesting that the event of HT itself is the primary determinant of poor prognosis.

**Figure 2:**
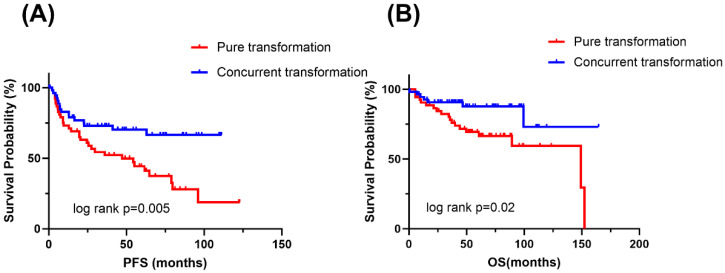
Survival outcomes stratified by transformation pattern. (**A**) PFS of patients with pure transformation (red) versus concurrent transformation (blue). Median PFS was 47.2 months in the pure transformation group; log-rank test *p* = 0.005. (**B**) OS of patients with pure transformation (red) versus concurrent transformation (blue). Hazard ratio for death was 2.56 (pure vs concurrent transformation); log-rank test *p* = 0.02.

In contrast, the transformation pattern provided meaningful risk stratification: patients with pure transformation experienced significantly worse PFS (median 47.2 months, *p* = 0.005) and OS (HR 2.56, *p* = 0.02) compared with those with concurrent transformation (Figure 2). In the PSM cohort, concurrent trDLBCL showed similar PFS to de novo DLBCL (*p* = 0.11), whereas three-group analysis indicated that the inferior PFS of the overall trDLBCL cohort was mainly driven by pure transformation. For OS, pure transformation showed only a nonsignificant trend toward worse survival compared with de novo DLBCL (*p* = 0.23), and OS did not differ significantly among de novo DLBCL, concurrent trDLBCL, and pure transformation (log-rank *p* = 0.56; Figure S1).

Within the pure transformation subgroup, the underlying indolent lymphoma subtype was relatively balanced, including 26 cases of transformed FL and 28 cases of transformed MZL. Survival outcomes were comparable between pure-FL and pure-MZL, with no significant differences in either PFS or OS (PFS, log-rank *p* = 0.99; OS, log-rank *p* = 0.23) (Figure S2). These results suggest that the adverse outcome associated with pure transformation was unlikely to be solely attributable to an imbalance in the underlying histological subtype.

### 3.4. Failure Patterns

Finally, to understand the clinical implications of transformation on early disease control, we analyzed patterns of treatment failure, including POD24 and CNS involvement. In the matched cohort, the incidence of POD24 was higher in trDLBCL than in de novo DLBCL (30.56% vs. 18.52%; OR 1.94, 95% CI: 1.05–3.56). This elevated rate of early progression accounts for the rapid decline in the PFS curve among trDLBCL patients, suggesting that the transformation background is associated with a greater likelihood of primary drug resistance.

Meanwhile, the incidence of secondary CNS involvement was low in both groups (2.78% in trDLBCL vs. 0.93% in de novo DLBCL, *p* = 0.62). These results indicate that although HT compromises systemic disease control, it does not substantially alter the CNS tropism of tumor cells, implying that intensified CNS-directed prophylactic strategies may not be necessary during initial treatment solely based on transformation history.

## 4. Discussion

In this large single-center cohort of over 1700 DLBCL patients, HT was identified as an independent adverse prognostic factor for PFS in treatment-naïve DLBCL. Notably, despite a lower incidence of bulky disease (≥7 cm) in trDLBCL patients (13.6% vs. 22.2%), these patients experienced significantly inferior PFS, highlighting that factors beyond tumor burden contribute to poor outcomes. This finding underscores the aggressive biological behavior of transformed tumors, potentially driven by clonal evolution and intrinsic therapy resistance [10,11,16,19,21,22].

Population-based analyses by Vaughn et al. (Biomarker Research) and Li et al. (Cancer) indicated that trDLBCL significantly impairs OS [6,10,17]. In contrast, Ghesquières et al. (JCO) reported that in the rituximab era, the OS difference between transformed and de novo DLBCL has narrowed or even disappeared [15]. Our results are consistent with prior studies in the rituximab era, and OS differences between transformed and de novo DLBCL have narrowed, whereas PFS remains compromised. To further delineate the source of this risk, we performed a three-arm comparison among De novo DLBCL, Concurrent trDLBCL, and Pure transformation. Our analysis confirmed that the inferior PFS in the overall trDLBCL cohort was primarily driven by the “pure transformation” subgroup, which exhibited markedly inferior PFS compared to both De novo and Concurrent groups. These findings may have therapeutic implications. The relatively similar outcomes between concurrent trDLBCL and de novo DLBCL suggest that established DLBCL-based treatment principles remain appropriate for concurrent trDLBCL. In contrast, the inferior PFS and higher risk of early progression observed in pure transformation indicate that this subgroup may require closer monitoring and risk-adapted therapeutic consideration. However, given the retrospective nature of this study and the absence of a significant OS difference, our data do not support a definitive recommendation for routine treatment intensification at present. Future prospective studies are warranted to determine whether intensified frontline therapy, consolidation, maintenance therapy, or earlier incorporation of novel agents, including chimeric antigen receptor T-cell (CAR-T) therapy, bispecific antibodies (BsAbs), and antibody–drug conjugates (ADCs; e.g., polatuzumab vedotin), could improve disease control in patients with pure transformation [23,24,25,26,27,28,29,30].

With regard to the influence of the original indolent subtype (t-FL vs. t-MZL), no significant survival difference was observed, which contrasts with findings by John L.Vaughn et al. (Blood Cancer Journal), who reported a poorer prognosis for t-MZL [5]. Notably, t-FL and t-MZL were relatively balanced in our cohort. Although transformation from FL is more commonly reported in many Western series, this distribution may reflect regional disease patterns, the treatment-naïve design of our study, and institutional referral characteristics. Previous Chinese or selected institutional studies have also reported that MZL-associated transformation can account for a substantial proportion of transformed lymphoma cases [5,15,16,31,32]. Therefore, the t-FL/t-MZL distribution in our single-center cohort should be interpreted cautiously and should not be generalized to all transformed lymphoma populations. In contrast, the transformation pattern provided meaningful risk stratification: patients with pure transformation had markedly inferior PFS and OS compared with those exhibiting concurrent transformation, identifying a clinically and biologically high-risk subgroup [11,14,15].

Failure pattern analysis revealed that the PFS disadvantage in trDLBCL is primarily driven by a higher incidence of POD24 (30.56% vs. 18.52%). However, the preservation of OS suggests a significant “catch-up effect,” attributable to both the high sensitivity of treatment-naïve disease to second-line regimens and autologous stem cell transplantation (ASCT), as well as increasing access to novel therapeutic agents. The advent of CAR-T, BsAbs, and ADCs has provided highly effective salvage options for trDLBCL patients with early progression, effectively closing the survival gap [23,24,25,26,27,28]. Conversely, secondary CNS involvement was low and comparable between groups (2.78% vs. 0.93%, *p* = 0.62), suggesting that HT does not substantially alter CNS tropism, and routine CNS prophylaxis should be guided by standard risk factors rather than transformation history alone [33,34].

Several limitations should be acknowledged in the current study. First, as a single-center retrospective study, sample sizes for rare subtypes and transformation patterns were limited, reducing statistical power to detect subtle prognostic differences. Second, comprehensive molecular profiling—including MYC, BCL2, and BCL6 rearrangements—was not available for all patients, limiting mechanistic interpretation, particularly for the pure transformation subgroup [19,21,22,35]. Third, detailed analyses of higher extranodal burden, such as more than three extranodal sites, and specific CNS high-risk extranodal sites, including adrenal, renal, testicular, or breast involvement, were not performed in the present study, as these variables were not predefined in the current analysis. Future studies incorporating systematic CNS-IPI-related variables are warranted to better clarify site-specific CNS relapse risk in trDLBCL. Finally, although PSM minimized baseline imbalances, residual confounding from unmeasured variables cannot be entirely excluded.

## 5. Conclusions

In summary, treatment-naive trDLBCL is associated with inferior PFS, primarily driven by early progression (POD24), whereas OS remains largely preserved due to effective salvage strategies. Pure transformation appeared to define a higher-risk subgroup with inferior disease control, supporting the need for future prospective studies to evaluate risk-adapted frontline, consolidation, or maintenance strategies.

## Figures and Tables

**Table 1:** Baseline characteristics of patients with trDLBCL and de novo DLBCL in the full cohort.

Characteristics	All (n = 1735)	Tr DLBCL (n = 118)	De Novo DLBCL (n = 1617)	*p*
Sex, n (%)				0.13
Female	751 (43.3)	59 (50.0)	692 (42.8)	
Male	984 (56.7)	59 (50.0)	925 (57.2)	
Age (years)	57.6 (14.4–89.2)	55.1 (14.6–86.7)	57.6 (14.4–89.2)	0.13
>60 years	728 (42.0)	41 (34.7)	687 (42.5)	
Ann Arbor stage, n (%)				0.10
I–II	803 (46.3)	46 (39.0)	757 (46.8)	
III–IV	932 (53.7)	72 (61.0)	860 (53.2)	
B symptoms, n (%)	504 (29.0)	32 (27.1)	472 (29.2)	0.63
Extra-nodal sites > 1, n (%)	618 (35.6)	40 (33.9)	578 (35.7)	0.69
Bulky disease, ≥7 cm, n (%)	375 (21.6)	16 (13.6)	359 (22.2)	0.03
ECOG performance status, n (%)				0.37
0–1	1628 (93.8)	113 (95.8)	1515 (93.7)	
>1	107 (6.2)	5 (4.2)	102 (6.3)	
LDH > normal, n (%)				0.43
>Normal	735 (42.4)	44 (37.3)	691 (42.7)	
Normal	935 (53.9)	68 (57.6)	867 (53.6)	
Unknown	65 (3.7)	6 (5.1)	59 (3.6)	
β2-microglobulin elevated, n (%)				0.901
>Normal	388 (22.4)	25 (21.2)	363 (22.4)	
Normal	1189 (68.5)	83 (70.3)	1106 (68.4)	
Unknown	158 (9.1)	10 (8.5)	148 (9.2)	
Cell of origin by Hans, n (%)				0.07
GCB	584 (33.7)	51 (43.2)	533 (33.0)	
Non-GCB	1096 (63.2)	63 (53.4)	1033 (63.9)	
Unknown	55 (3.2)	4 (3.4)	51 (3.2)	
Transformation histology, n (%)				<0.01
t-MZL		56 (47.5)		
t-FL		55 (46.6)		
others		7(5.9)		
Transformation pattern, n (%)				0.58
Pure transformation		56 (47.5)		
Concurrent transformation		62 (52.5)		

**Table 2:** Univariate and multivariate Cox regression analysis for progression-free survival.

Variables	Univariate Analysis			Multivariate Analysis		
	HR	95% CI	*p* Value	HR	95% CI	*p* Value
Sex	1.136	0.969–1.333	0.115			
Age (years), median	1.374	1.173–1.609	<0.001			
Ann Arbor stage	3.057	2.559–3.652	<0.001	1.820	1.390–2.383	<0.001
B symptoms	1.769	1.504–2.081	<0.001			
Extra-nodal sites > 1	2.452	2.094–2.872	<0.001	1.503	1.188–1.903	<0.001
Bulky disease, ≥7 cm	1.878	1.578–2.234	<0.001			
ECOG performance status	3.216	2.485–4.162	<0.001	2.063	1.532–2.779	<0.001
LDH > normal	2.762	2.331–3.272	<0.001	1.833	1.510–2.226	<0.001
β2-microglobulin elevated	2.149	1.792–2.577	<0.001			
Non-GCB	1.398	1.172–1.668	<0.001	1.279	1.048–1.561	0.015
trDLBCL	1.447	1.101–1.900	0.012	1.754	1.297–2.372	<0.001
Pathology						
Double-hit	1.511	0.853–2.675	0.184			
Double-expressor	1.422	1.172–1.725	<0.001	1.265	1.017–1.573	0.035
High-grade NOS	2.140	1.108–4.133	0.044			

Factors with *p* < 0.1 in the univariate analysis were subjected to multivariate analysis afterwards. Forward stepwise Cox proportional-hazard modeling was used in the multivariate analysis of risk factors.

## Data Availability

Any data in this study are available from the corresponding authors on reasonable request.

## Supplementary Materials

The following supporting information can be downloaded at: https://www.mdpi.com/article/10.3390/cancers18101641/s1. Figure S1: Survival Outcomes According to Transformation Pattern in the Matched Cohort; Figure S2: Survival outcomes according to underlying indolent lymphoma subtype within the pure transformation subgroup.
