# Supplementary material for: Clinical Outcomes of Treatment-Naive Transformed vs. De Novo Diffuse Large B-Cell Lymphoma: A Propensity Score-Matched Analysis of 1735 Cases
Source: Cancers (Basel). 2026 May 19;18(10):1641. doi: 10.3390/cancers18101641 (PMC13205124; doi:10.3390/cancers18101641)
Supplement: Supplementary file 1 [file cancers-18-01641-s001.zip › cancers-4295028-supplementary.pdf]

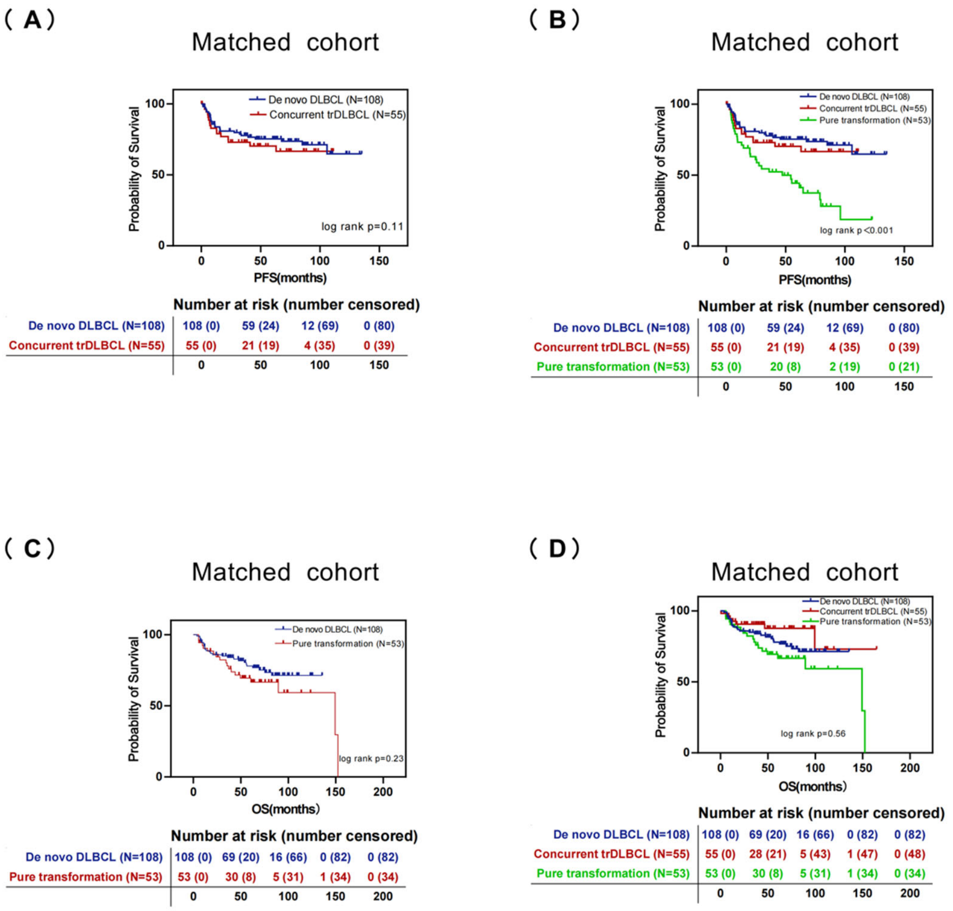

Figure S1. Survival Outcomes According to Transformation Pattern in the Matched Cohort. (A) Kaplan–Meier curves comparing PFS between de novo DLBCL and concurrent trDLBCL in the matched cohort. No significant difference in PFS was observed between the two groups (log-rank P = 0.11). (B) Kaplan–Meier curves comparing PFS among de novo DLBCL, concurrent trDLBCL, and pure transformation in the matched cohort. The three-group comparison showed a significant difference in PFS (log-rank P < 0.001), with pure transformation showing inferior PFS compared with the other two groups. (C) Kaplan–Meier curves comparing OS between de novo DLBCL and pure transformation. No significant difference in OS was observed between the two groups (log-rank P = 0.23). (D) Kaplan–Meier curves comparing OS among de novo DLBCL, concurrent trDLBCL, and pure transformation. OS did not differ significantly among the three groups (log-rank P = 0.56).

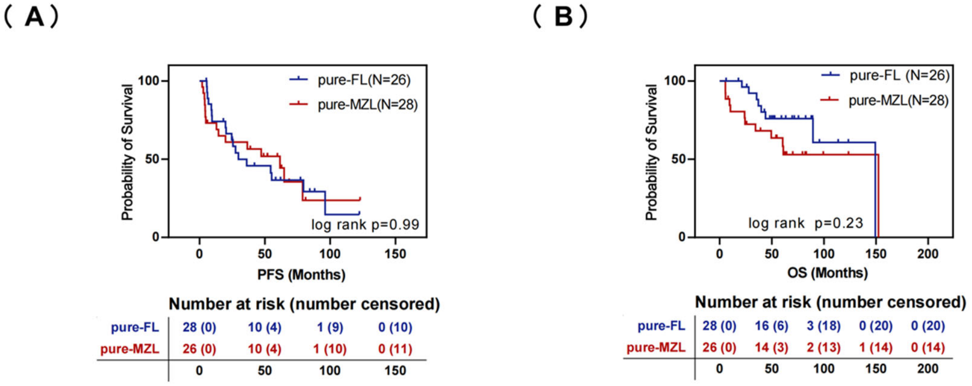

Figure S2. Survival outcomes according to underlying indolent lymphoma subtype within the pure transformation subgroup. (A) PFS comparison between pure-FL and pure-MZL. No significant difference in PFS was observed between the two groups (log-rank P = 0.99). (B) OS comparison between pure-FL and pure-MZL. No significant difference in OS was observed between the two groups (log-rank P = 0.23).
